# NT-pro-BNP as a Predictor for Recurrence of Atrial Fibrillation after Primary Cryoballoon Pulmonary Vein Isolation

**DOI:** 10.3390/jcm11247400

**Published:** 2022-12-14

**Authors:** Hermann Blessberger, Thomas Lambert, Alexander Nahler, Denis Hrncic, Simon Hönig, Julian Maier, Stefan Rechberger, Armin Windhager, Christian Reiter, Joerg Kellermair, Juergen Kammler, Helga Wagner, Clemens Steinwender

**Affiliations:** 1 Department of Cardiology, Kepler University Hospital, Medical Faculty, Johannes Kepler University Linz, Krankenhausstrasse 9, 4021 Linz, Austria; 2 Department of Internal Medicine II, Paracelsus Medical University, 5020 Salzburg, Austria; 3 Institute of Applied Statistics, Johannes Kepler University Linz, 4040 Linz, Austria

**Keywords:** atrial fibrillation, pulmonary vein isolation, cryoballoon, NT-pro-BNP, success rate, long-term follow-up

## Abstract

NT-pro-BNP is produced in the cardiac atria and ventricles in response to increased wall stress. It may be a marker of both AF disease progression and co-morbidities that affect success after pulmonary vein isolation (PVI). This single-center retrospective study analyzed the association between pre-procedural NT-pro-BNP serum levels and the long-term outcome after a first-ever PVI in cryo-technique. Patients were followed by searching the hospital information system and conducting structured telephone interviews. Treatment failure was defined as any relapse of atrial fibrillation (AF) occurring 90 days after the index PVI at the earliest. Kaplan–Meier survival curves and Cox proportional hazards models were computed to assess the impact of NT-pro-BNP on AF recurrence. Following 374 patients over a median of 3.8 years (range: 0.25–9.4 years), baseline NT-pro-BNP was associated with the combined endpoint in univariate analysis (HR 1.04 per 100 pg/mL increase, 95% CI: 1.02–1.07, *p* < 0.001). Results were virtually unchanged in the multivariate model or if the data were log-transformed. Intraprocedural left atrial pressure correlated positively with log NT-pro-BNP. NT-pro-BNP was associated with AF relapse during a long-term follow-up after first-ever cryo-PVI in our cohort of patients with predominantly normal left ventricular function. This lab parameter is easy to obtain and has significant potential to guide treatment decisions.

## 1. Introduction

The number of people affected by atrial fibrillation is steadily increasing and was estimated at 37 million worldwide in 2017 [1]. Therefore, efficacious and cost-efficient treatment strategies are critical to combat this pandemic. Catheter ablation—as opposed to medical antiarrhythmic therapy—has been shown to be superior in preserving sinus rhythm in symptomatic patients with atrial fibrillation (AF) [2]. It helps relieve symptoms and improve quality of life and may even benefit the cardiovascular outcome in patients with heart failure [3]. When considering catheter ablation for the treatment of AF, pulmonary vein isolation (PVI) is the procedure of choice as endorsed by current guidelines [4]. The ‘Fire and Ice’ trial proved that PVIs performed using cryo-energy are non-inferior to those performed with radiofrequency energy in patients with paroxysmal AF [5]. Irrespective of the energy source used, patients after PVI face a 12-month success rate between 65% and 85% [6,7], whereas the risk of potentially life-threatening complications is about 2–3% [8]. Hence, there is a great need for selection criteria in everyday practice to determine which patients will benefit most from a PVI. This would allow for a patient-tailored approach that maximizes the net clinical benefit. The enzymatic splitting of proBNP generates NT-pro-BNP and BNP in a 1:1 fashion. The physiologically inactive peptide NT-pro-BNP has a longer half-life than its active counterpart BNP and is produced by both the cardiac atria and ventricles in response to increased myocardial wall stress. Under physiological conditions, about 30% of serum NT-pro-BNP is released from the atria [9]. In addition to its importance in the diagnosis and treatment monitoring of heart failure, NT-pro-BNP is also released during AF due to asynchronous contraction of the atrial myocardium with tethering of surrounding tissue [10]. NT-pro-BNP may thus be a potential marker of AF disease progression (e.g., arrhythmic burden and extent of atrial cardiomyopathy) or co-morbidities (such as heart failure) that may have an impact on the success rate after PVI [11]. This study evaluates the prognostic value of pre-procedural NT-pro-BNP for the outcome after a first single cryo-PVI in patients with paroxysmal or persistent AF.

## 2. Materials and Methods

### 2.1. Patient Cohort and Baseline Data Collection

We conducted a single-center retrospective observational cohort study to analyze the association between pre-procedural NT-pro-BNP serum levels and the recurrence rate of AF after the first-ever PVI in cryo-technique. Local ethics committee approval of the study protocol was sought and granted. The study was conducted in accordance with the Declaration of Helsinki. Procedural and baseline data were retrieved from our institution’s Atrial Fibrillation Cryo Ablation Registry (AFCAR) and the electronic hospital information system. Cases were included if patients were (1) at least 18 years old when the PVI was performed and (2) had undergone their first-ever PVI in cryo-technique to treat paroxysmal or persistent AF at least three months before. Cases were excluded if (1) it had been a re-do procedure (i.e., any prior pulmonary vein isolation regardless of the technique used) or (2) no pre-procedural NT-pro-BNP values were available during the index stay. Blood samples for NT-pro-BNP measurements were drawn during the index stay before the pulmonary vein isolation was performed, usually at admission to the hospital on the day before the procedure. The data collected comprised demographic variables, routine lab parameters, co-morbidities, and procedural data (such as type of atrial fibrillation, left atrial pressure (LAP), fluoroscopy and procedure time, and complications during the index hospital stay).

### 2.2. Cryo-Pulmonary Vein Isolation

All patients received a pre-procedural cardiac computed tomography to determine the suitability of the pulmonary vein anatomy for cryoablation and a transesophageal echocardiography to rule out a thrombus in the left atrium or the left atrial appendage. Only large common ostia that would have made adequate occlusion by the cryoballoon impossible were considered an exclusion criterion for not performing a cryo-ablation. Invasive blood pressure and percutaneous oxygen saturation were continuously monitored. After initiation of deep sedo-analgesia with propofol (2%) and fractionated fentanyl and placing of the diagnostic catheters (coronary sinus: 5 French decapolar Supreme CSL^®^, His: 4 French quadripolar Supreme^®^, Abbott Medical SA, Wavre, Belgium), unfractionated heparin was administered (100 I.U. per kg body weight). The transseptal puncture was then performed using an SL-0 or SL-1 sheath (Abbott Medical SA, Belgium) and a BRK-1 Brockenbrough needle (Medtronic Inc., Minneapolis, MN, USA). Fluoroscopy, contrast, and pressure monitoring were used for guidance. The minimum and maximum left atrial pressures were measured after removing the needle and flushing the sheath with saline. A temperature probe (SensiTherm^®^, Abbott Medical SA, Belgium) was inserted into the esophagus, and cryo-energy was immediately stopped if the temperature fell below 15 °C. Activated clotting time was measured every 20 min and targeted at 300–350 s. After the introduction of the FlexCath^®^/FlexCath Advance^®^ sheath (Medtronic Inc., Minneapolis, MN, USA) in the left atrium via a J-wire, the selective angiography of all four pulmonary veins was performed using the FlexCath^®^ sheath. Then, the antral PVI of all four veins was accomplished with a cryoballoon system and a circular mapping catheter (28 mm 1st, 2nd, or 3rd generation Arctic Front^®^/Arctic Front Advance^®^ cryoballoon and Achieve^®^/Achieve Advance^®^ circular mapping catheter, Medtronic Inc., Minneapolis, MN, USA). The complete occlusion of pulmonary veins was visualized by using contrast and recording an adequate temperature drop. Vein isolation during the freeze with loss of pulmonary vein signals was visualized via the circular mapping catheter, if possible. A 23 mm cryoballoon or cryo-tip focal touch-up (Freezor Max^®^, Medtronic Inc., Minneapolis, MN, USA) could be used at the operator’s discretion if deemed necessary. When isolating the right lower and upper pulmonary veins, the phrenic nerve was stimulated via the His catheter placed in the superior vena cava. Phrenic nerve function was monitored by tactile feedback of diaphragmatic contraction. If response to stimulation decreased, the delivery of cryo-energy was immediately stopped. Cryo-energy was dosed depending on the generation of the system (1st generation: 1 freeze of 240 s + 1 bonus freeze of 240 s, 2nd/3rd generation: time to isolation + 120 s). After application of the freezes, exit and entry blocks were confirmed. If the waiting time was less than 30 min, 15 mg of adenosine were administered i.v. to identify dormant conduction.

### 2.3. Definition of Treatment Failure

The combined endpoint was defined as any relapse of AF. To qualify for this outcome, which was considered a treatment failure, patients had to meet at least one of four criteria after a blanking period of 90 days after the procedure: AF or atrial flutter or atrial tachycardia lasting more than 30 s that was (1) documented on a 12-lead ECG or (2) required electrical or medical cardioversion or (3) required hospital admission or (4) required a re-do PVI. Furthermore, a re-do PVI was always considered a failure, even if it was completed within 90 days after the initial index cryo-PVI. In this case, the failure date was set at 90 days after the index procedure. Patients reached the endpoint with the first occurrence of any of these four criteria or were censored at the time of their last follow-up.

### 2.4. Follow-Up and Endpoint Data Collection

A routine follow-up with a 12-lead ECG and a detailed history regarding arrhythmogenic complaints was performed in all patients in our outpatient clinic after three and 12 months. A 24 h Holter ECG was recorded after three months. Patients received additional 12-lead ECGs and Holter recordings in our clinic when they contacted the staff because of symptoms suspicious of atrial fibrillation or reported them to their primary care physician or referring cardiologist. Upon discharge from the index stay, antiarrhythmic drugs were routinely continued until three months after the procedure and then stopped if no AF relapse was documented. An exception to this rule was beta-blocker treatment in individuals without AF recurrence at three months, in whom the drug could be continued at the physician’s discretion. Structured follow-up data collection was conducted between 2018 and 2019. As a first step, the patients’ survival status was retrieved from local registration authorities. In a second step, follow-up data were collected for each patient (1) by searching the hospital information system and (2) conducting a telephone interview. During this interview, a structured questionnaire was administered to the patient by a trained study team member. Items listed in the questionnaire included the occurrence of any arrhythmia since the index PVI procedure (ECG documentation, cardioversion, hospital admission, or re-do procedure), current arrhythmic symptoms and antiarrhythmic medication. Discharge letters or medical reports were requested if the event did not occur at our center. An invitation letter was sent to patients with whom no telephone contact could be established, asking them to respond to it or call a study team member. Finally, if patients were alive and this attempt was unsuccessful, their primary care physician and next of kin were contacted. Patients were considered lost to follow-up if no information could be obtained through all these steps. Data are reported according to the STROBE statement for cohort studies [12].

### 2.5. Statistical Methods

Categorical data are presented as counts and percentages. The normal distribution of numeric data was assessed by visual inspection and formally tested by the Shapiro–Wilk test. Numeric data are given as mean and standard deviation or described as median, range, and interquartile range (range from the 25th to the 75th percentile), as appropriate. Kaplan–Meier survival analysis with a log-rank test was used to assess the impact of pre-procedural NT-pro-BNP levels on AF recurrence. Clinically important effect modifiers were investigated using multivariate Cox proportional hazards models. Especially age, sex, BMI, renal function, and left ventricular function were factors shown to influence NT-pro-BNP serum levels and were thus adjusted for [13]. A time-dependent ROC analysis with 0/1 nearest-neighbor kernel smoothing of the conditional survival function was calculated. A specific NT-pro-BNP cut-off was estimated applying the method described by Liu et al. [14]. P-spline models were generated to assess the possibility of non-linear effects of continuous variables. The association between NT-pro-BNP levels and LAP was analyzed by applying Pearson’s correlation and a linear regression model. As this was a hypothesis-generating study, *p*-values were not adjusted for multiple testing. Calculations were performed with R Studio version 4.1.2 (http://cran.r-project.org/) and Intercooled STATA (release 14.0, StataCorp LP, College Station, TX, USA).

## 3. Results

### 3.1. Baseline Data of the Cohort under Investigation

We could identify 404 patients in whom a first-ever PVI in cryo-technique was performed at our center between February 2009 and July 2017 and for whom a pre-procedural baseline NT-pro-BNP value during the index stay was available. Of these, 29 patients (7.2%) were excluded from the analysis due to the following reasons: Refusal to participate in the study and the telephone interview (n = 4), loss to follow-up but alive (n = 21 out of 28 subjects were excluded in whom no pre-defined endpoint was documented in the hospital information system), patients who died and were thus lost to follow-up (nine patients died during follow-up, of whom follow-up data were unavailable for n = 4 subjects). Death was not considered an endpoint for this study. Further analyses were performed in the 375 remaining patients. Baseline data of the cohort are shown in Table 1. Applying a univariate Cox proportional hazards model, BMI, type of atrial fibrillation, left ventricular ejection fraction, LAP during the procedure, beta-blocker use at discharge, left atrial volume, and NT-pro-BNP serum levels were associated with the combined endpoint at follow-up.

### 3.2. Procedural Data

At the time of the index PVI, 276 patients were in sinus rhythm (73.6%), 75 (20%) were in atrial fibrillation, and 6 (1.6%) were in right atrial flutter. In 18 subjects (4.8%), data on rhythm at baseline were missing. Ninety-six patients (25.6%) were treated with a first-generation, 247 (65.9%) were treated with a second-generation and 32 (8.5%) were treated with a third-generation (“short tip”) cryoballoon. The larger 28 mm diameter balloon was applied for all pulmonary vein isolations except for 44 cases (11.7%: application of the smaller 23 mm balloon in 18 cases, and of both balloon types in 26 procedures). A touch-up cryo-tip catheter was used in 14 (3.7%) cases (in eight first-generation, five second-generation, and one third-generation PVIs). The median total freezing time was 19 min (IQR: 16–25 min), and fluoroscopy time was 19 min (IQR: 13–30 min). The total procedure time was 90 min (IQR: 70–130 min). Procedural success—as defined by isolation of all pulmonary veins—was achieved in all but 13 individuals (3.5%). Periprocedural complications occurred in 46 patients (12.3%): 6 (1.6%) vagal responses during ablation, 41 (10.9%) transient right-sided phrenic nerve palsies (none of which persisted after 12 months), 23 (6.1%) mild pericardial effusions not requiring pericardiocentesis that were interpreted as inflammatory responses (including 2 (0.5%) cases of post-procedural pericarditis), 1 (0.3%) cardiac tamponade requiring immediate pericardiocentesis, 4 (1.1%) transient ischemic attacks after the procedure, 11 (2.9%) groin complications (7 (1.9%) hematomas, 1 (0.3%) AV fistula, 3 (0.8%) spur aneurysms), and 1 (0.3%) postprocedural death. No pneumothoraces or atrio-esophageal fistulas occurred. Antithrombotic therapy of patients at discharge included a direct oral anticoagulant (DOAC) in 159 (42.5%), a DOAC plus single antiplatelet therapy in 7 (1.9%), phenprocoumon in 194 (51.9%), phenprocoumon plus single antiplatelet therapy in 10 (2.7%), low-molecular-weight heparin in 2 (0.5%), and triple therapy (acetylsalicylic acid + clopidogrel + phenprocoumon) in 2 (0.5%) cases.

### 3.3. Follow-Up and Time to Event Analysis

**Figure 1:**
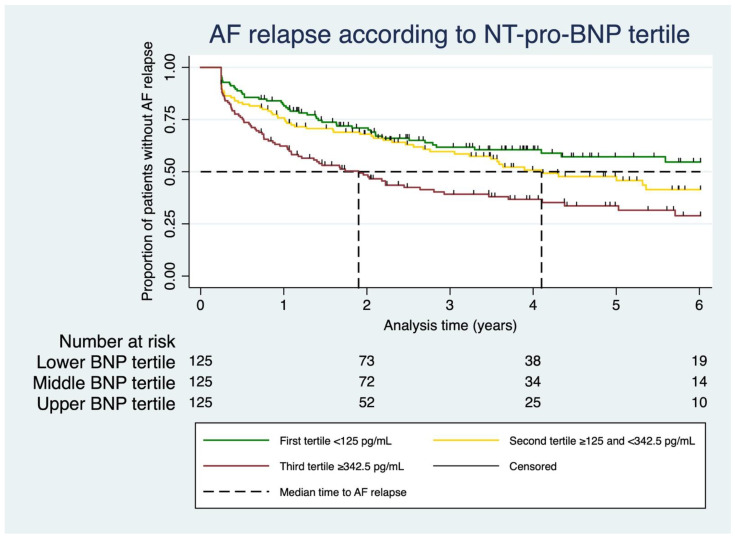
Atrial fibrillation relapse according to NT-pro-BNP tertile. Kaplan–Meier analysis of AF relapse according to pre-procedural NT-pro-BNP tertile. Dashed line = median time to AF relapse. Small vertical marks on the Kaplan–Meier curves indicate censored observations.

Patients were followed over a median of 3.8 years (IQR: 2.2–6.0 years, range: 3.0 months to 9.4 years). One patient out of 375 was excluded from the time-to-event analysis, as he died shortly after the procedure within the index stay and was hence not at risk for AF relapse. Of the remaining 374 patients, nine patients (2.4%) died during the follow-up. Death was not considered an outcome of interest and was not related to AF or the index procedure in all cases except the one case mentioned above that was excluded from the time-to-event analysis (see ‘Procedural data’ above). Of the entire cohort, 189 subjects (50.5%) met at least one component of the combined endpoint after the three-month blanking period: n = 176 (47.1%) ECG-documented atrial arrhythmia, n = 68 (18.2%) medical or electrical cardioversion due to AF relapse, n = 106 (28.3%) re-do pulmonary vein isolation, and n = 130 (34.8%) any hospitalization because of AF. In a univariate Cox proportional hazards model, the risk of an AF relapse increased with higher NT-pro-BNP serum levels (HR 1.04 per 100 pg/mL increase, 95% CI: 1.02–1.07, *p* < 0.001). When splitting the NT-pro-BNP values into tertiles, the gradual increase in this risk with higher levels could be observed particularly well (Figure 1, 1st tertile: NT-pro-BNP < 125 pg/mL, 2nd tertile: ≥125 and <342.5 pg/mL, 3rd tertile: ≥342.5 pg/mL). In a univariate Cox proportional hazards model, the risk of relapse was twice as high in the third tertile compared with the first (2nd vs. 1st tertile: HR 1.39, 95% CI: 0.95–2.03, *p* = 0.086; 3rd vs. 1st tertile: HR 2.10, 95% CI: 1.46–3.01, *p* < 0.001). As the NT-pro-BNP values were not normally distributed, the data were also log-transformed (natural logarithm). The calculations with these logarithmized (log) NT-pro-BNP values again yielded a similar association with the combined endpoint (HR 1.31 per increase of one log unit of NT-pro-BNP, 95% CI: 1.16–1.49, *p* < 0.001).

### 3.4. Failure Curves Derived from Our Data

**Figure 2:**
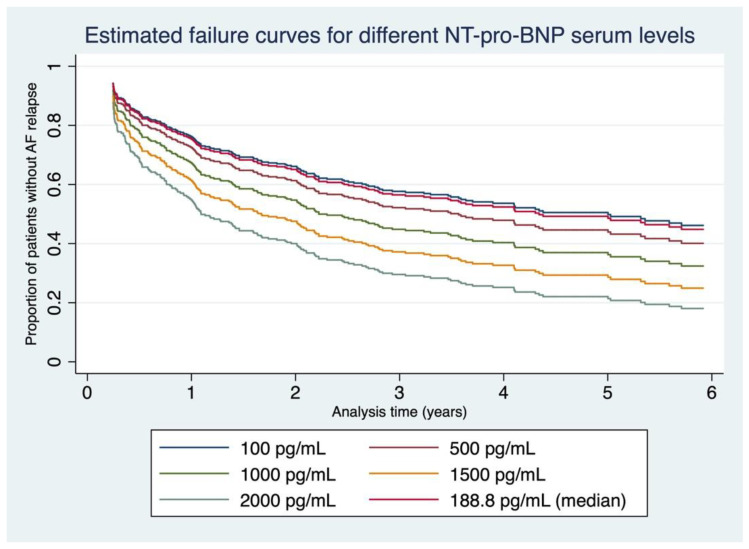
Estimated failure curves for different NT-pro-BNP serum levels. Different failure curves derived from our data depicting estimated AF relapses for the first six years after the first-ever cryo-PVI for baseline NT-pro-BNP levels between 100 and 2000 pg/mL.

Based on our data set, we constructed failure curves for different baseline NT-pro-BNP serum levels (between 100 and 2000 pg/mL) for the first six years after the first-ever PVI in cryo-technique (Figure 2). A time-dependent ROC analysis calculated for procedural success at 6 years identified an NT-pro-BNP value of 166.7 pg/mL as an optimized cut-off obtained by maximizing the concordance probability [14] and yielded an AUC of 0.638 (sensitivity 62.9%, specificity 61.1%, for ROC curve, see Supplementary Figure S1).

### 3.5. Multivariate Cox Regression Models

Adjusting for important clinical confounders, the association between NT-pro-BNP serum levels and AF relapses was also assessed by applying multivariate Cox proportional hazards models (Table 2). The results proved to be robust to potential confounding and remained virtually unchanged. In addition to NT-pro-BNP levels, non-paroxysmal AF status, female sex, and greater left atrial volume were associated with AF relapse in multivariate models. To allow for the potential non-linearity of effects, we estimated the effects of continuous covariates via P-splines. The non-linear part of the effect is significant only for NT-pro-BNP. Plots of the estimated effects with 95% confidence intervals are provided in Supplementary Figure S2.

### 3.6. Association between NT-pro-BNP Levels and LAP

**Figure 3:**
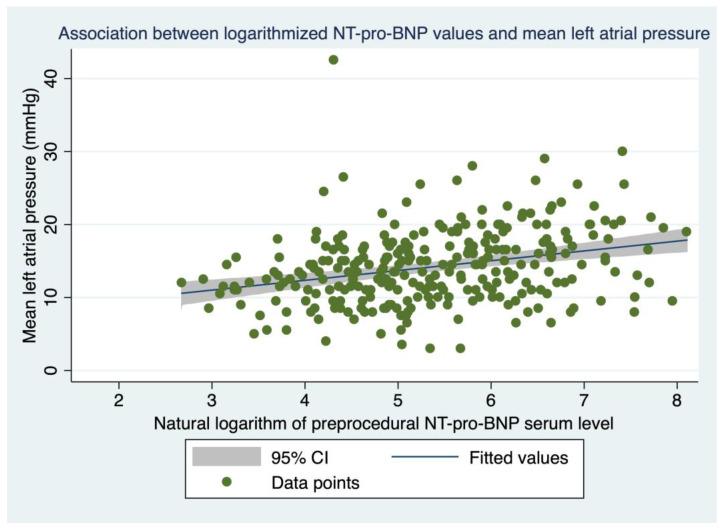
Association between logarithmized NT-pro-BNP values and mean left atrial pressure. Association between pre-procedural log NT-pro-BNP values and mean LAP measured during the index PVI. Blue line = regression line, gray zone = 95% confidence interval, green dots = individual data points.

Mean left atrial pressure, as assessed immediately after a transseptal puncture during the procedure, varied between 3.0 and 42.5 mmHg (median: 13.5, IQR: 11.0–17.0 mmHg). Readings were available for 278 (74.1%) individuals in our cohort. Linear regression analysis showed that pre-procedural log NT-pro-BNP values were associated with intra-procedural LAP (mean LAP rises by 1.34 mmHg with the increase of one log unit NT-pro-BNP, 95% CI: 0.83–1.86 mmHg, *p* < 0.001, Pearson’s correlation coefficient r = 0.295, Figure 3). This association was unaffected if the left ventricular ejection fraction, as a significant confounder, was introduced into the model (1.47 mmHg, 95% CI: 0.91–2.03 mmHg, *p* < 0.001). If only the subgroup of patients in sinus rhythm at the time of the index procedure (for whom a LAP reading was available) were considered for analysis (n = 201, 53.6% of the cohort), the association between LAP and log NT-pro-BNP remained unchanged (1.39 mmHg, 95% CI: 0.68–2.09 mmHg, *p* < 0.001, r = 0.264).

## 4. Discussion

In our study, we performed a retrospective long-term follow-up of 375 patients after their first-ever cryo-PVI, irrespective of the type of atrial fibrillation. A significant proportion of our cohort (50.5%) experienced some form of AF relapse during a median follow-up time of 3.8 years (range: 3 months to 9.4 years). Untransformed and logarithmized pre-procedural NT-pro-BNP serum levels were associated with the combined endpoint in a univariate and a multivariate Cox proportional hazards model that adjusted for important clinical confounders. Furthermore, log NT-pro-BNP values were also associated with intra-procedural mean LAP.

### 4.1. Procedural Variables and Long-Term Success Rate after the First-Ever Cryoballoon Ablation

As compared with the cryoablations performed in the ‘Fire and Ice’ trial, the procedure time was shorter in our cohort (median 90.0 min vs. average 124.4 min), whereas the fluoroscopy time and the success rate were within the same range (median 19.0 min vs. average 21.7 min, and 96.5% vs. 98.9%, respectively) [7]. However, in contrast to this trial, we also included patients with persistent or long persistent atrial fibrillation (12.8% of the study cohort). Patients were, on average, about the same age (59 years), more likely to be male (71% vs. 59%), and had a lower burden of co-morbidities as reflected by the CHADS_2_VA_2_Sc score (median 1.0 vs. average 1.9). The complication rate was within the range reported in the literature for patients undergoing cryo-pulmonary vein isolations [15]. In general, reports of long-term outcomes after more than three years after PVI are rare. It must also be noted that these reported success rates heavily depend on the precise mode of follow-up (‘conventional’ follow-up with 12-lead ECGs, Holter ECGs, and telephone interviews vs. implantable cardiac monitors) as well as the inclusion of results after re-do procedures [16]. Furthermore, a steady decrease in freedom from AF after the PVI procedure over time must be assumed. Our long-term success rate of about 50% after a median follow-up of 3.8 years was assessed ‘conventionally’. Our results are comparable to a randomized controlled trial that studied the outcome after the first-ever cryo-PVI in patients with paroxysmal atrial fibrillation, also used a ‘conventional’ follow-up approach, and showed an AF-free survival of 47% after five years [17]. The impact of also considering AF-free survival after a re-do procedure is underscored by the fact that the five-year success rate rose to 75% in this very same cohort if patients with re-do procedures were not classified as ultimate failures and re-included in the analysis.

### 4.2. NT-pro-BNP as a Predictor of Success after Cryo-PVI

The association of cryo-PVIs with a certain failure and complication rate as well as radiation exposure calls for a careful selection of individuals who will benefit most from this procedure (‘patient-tailored approach’). In this regard, a simple predictor of ablation success, such as NT-pro-BNP, would be of great value to adequately educate patients about their individual chances of success. This seems especially important during the shared decision-making process before the ablation. A single blood draw may help estimate the approximate success rate in an outpatient setting. In our patient cohort, we could show that pre-procedural NT-pro-BNP serum levels were independently associated with long-term success after PVI in a graded manner. These results are in line with the findings of two meta-analyses comprising nine trials [18,19]. In contrast to our investigation, radiofrequency energy was used for pulmonary vein isolation in all these studies. Up to now, data on the predictive value of NT-pro-BNP after cryo-PVI remain sparse. In a study that exclusively recruited 26 patients who underwent a cryo-PVI, no difference in baseline NT-pro-BNP levels could be identified between patients with and without AF relapse during a follow-up period of six months [20]. The NT-pro-BNP cut-off of 167.3 pg/mL determined in our ROC analysis was slightly lower compared with the studies by Hwang et al. (220 pg/mL) and the SMAC-AF sub-study (280 pg/mL) [21,22]. This finding might be explained by a shorter follow-up time in both these studies (three months and 24 months, respectively, vs. 3.8 years in our trial), variations caused by a lower number of cases, or the use of radiofrequency energy as opposed to cryo-energy. These cut-off values are in the range of the upper limit of normal as assessed by the 97.5th quintile in a sample of the Framingham Heart Study Generation 3 cohort for males and females aged 55–59 years (106.4 pg/mL and 215.9 pg/mL, respectively) [13]. As NT-pro-BNP levels rise with age, these 97.5th quintiles were much higher in healthy adults aged 65 years and older (male: 663 pg/mL, female: 744 pg/mL) [23].

### 4.3. Association between NT-pro-BNP and LAP

**Figure 4:**
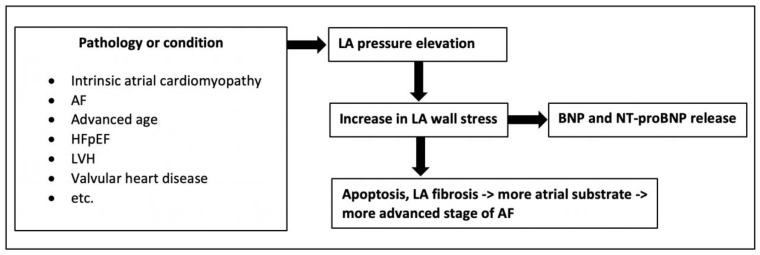
Pathophysiology of NT-pro-BNP serum levels and left atrial pressure. Association between mean LAP, pre-procedural NT-pro-BNP levels and AF disease progression [24,25,26]. AF = atrial fibrillation, HFpEF = heart failure with preserved ejection fraction, LVH = left ventricular hypertrophy, LA = left atrial, BNP = brain-type natriuretic peptide.

An elevation of LAP may result from various pathologies (Figure 4) [24]. It can be seen as an indicator of the extent of left atrial disease, the progression of AF disease, and the outcome after PVI. As LAP can only be measured invasively after the procedure has started, the decision to perform pulmonary vein isolation has already been made. Because of the moderate correlation between log NT-pro-BNP values and mean LAP, NT-pro-BNP measurements may help to non-invasively estimate LAP. This, in turn, may provide the pathophysiological basis to explain the association between baseline NT-pro-BNP levels and long-term outcome after cryo-PVI.

### 4.4. Limitations

This study had a single-center retrospective design with the limitations inherent in such. Furthermore, we conducted a ‘conventional’ follow-up to detect AF recurrences. The association between serum NT-pro-BNP levels and the combined endpoint representing any form of AF relapse and the calculated cut-offs have to be confirmed in a separate validation cohort. NT-pro-BNP levels might have been influenced by the level of physical activity which could not be determined in our cohort. Furthermore, our group of patients comprised only five (1.3%) individuals with heart failure with a reduced ejection fraction (below 40%). Therefore, the impact of a reduced left ventricular ejection fraction on this association remains to be determined, and our findings cannot be reliably applied to this subgroup. Obesity may cause falsely low NT-pro-BNP values. We accounted for this as BMI was included in the calculated multivariate Cox proportional hazards models as an effect modifier.

## 5. Conclusions

Our retrospective single-center trial found that pre-procedural NT-pro-BNP serum levels were associated with AF relapse during a long-term follow-up of median 3.8 years after a single first-ever cryo-PVI. This standard laboratory parameter is easy to obtain in daily clinical routine and has significant potential to guide patient-tailored treatment decisions. A correlation between the mean LAP and NT-pro-BNP in our patient cohort may be the pathophysiological explanation for these findings. NT-pro-BNP should be further investigated in prospective trials to confirm our findings.

## Figures and Tables

**Table 1:** Baseline characteristics of the cryo-PVI cohort (n = 375).

Parameter	Median (IQR) n (%) µ ± SD	Range	Missing n (%)	*p*-Value ^†^	HR (95% CI)
Age at PVI ^a^	59 (52–68)	21–81	0 (0.0)	0.109	1.01 (1.00–1.02)
Male sex ^b^	267 (71.2)	n/a	0 (0.0)	0.191	0.81 (0.60–1.11)
BMI (kg/m^2^) ^a^	27.1 (24.8–30.1)	16.1–50.9	0 (0.0)	0.011	1.04 (1.01–1.08)
Paroxysmal AF ^b^	327 (87.2)	n/a	0 (0.0)	n/a	
Persistent AF ^b^ (vs. paroxysm. AF)	43 (11.5)			0.005	1.81 (1.20–2.73)
Long-persistent AF ^b^ (vs. paroxysm. AF)	5 (1.3)			0.016	2.98 (1.22–7.28)
EF (%) ^a^	65 (60–65)	35–70	36 (9.6)	0.055	0.98 (0.95–1.00)
GFR (mL/min/1.73 m^2^) ^a^	78.3 (66.2–89.7)	6.5–139.7	1 (0.3)	0.659	1.00 (0.99–1.01)
TSH (µU/mL) ^a^	1.33 (0.96–2.11)	0.03–28.39	126 (33.6)	0.717	1.01 (0.94–1.09)
NT-pro-BNP (pg/mL) ^a^	188.8 (86–465)	5–3309	0 (0.0)	<0.001	1.00 (1.00–1.00)
log NT-pro-BNP (pg/mL) ^c^	5.3 ± 1.2	1.61–8.10	0 (0.0)	<0.001	1.31 (1.16–1.49)
Arterial hypertension ^b^	196 (52.3)	n/a	0 (0.0)	0.275	1.17 (0.88–1.56)
Hypercholesterolemia ^b^	253 (67.5)	n/a	0 (0.0)	0.224	0.83 (0.62–1.12)
Diabetes ^b^	29 (7.7)	n/a	0 (0.0)	0.388	0.78 (0.44–1.37)
Coronary heart disease ^b^	36 (9.6)	n/a	0 (0.0)	0.189	1.35 (0.86–2.10)
HFrEF (EF < 40%) ^b^	5 (1.3)	n/a	36 (9.6)	0.638	0.72 (0.18–2.88)
History of stroke ^b^	27 (7.2)	n/a	0 (0.0)	0.329	0.74 0.40–1.36
PAD ^b^	4 (1.1)	n/a	0 (0.0)	0.174	2.21 (0.71–6.93)
CHA_2_DS_2_VaSc score ^a^	1 (0–2)	0–5	36 (9.6)	0.193	1.08 (0.96–1.22)
RR systolic (mmHg) ^a^	130 (120–140)	90–200	0 (0.0)	0.689	1.00 (0.99–1.01)
RR diastolic (mmHg) ^c^	80 (70–80)	50–110	0 (0.0)	0.974	1.00 (0.99–1.01)
LAP maximum (mmHg) ^a^	18 (15–23)	6–55	97 (25.9)	0.004	1.04 (1.01–1.06)
LAP minimum (mmHg) ^a^	9 (6–12)	0–30	97 (25.9)	0.034	1.04 (1.00–1.08)
Mean LAP (mmHg) ^a^	13.5 (11.0–17.0)	3.0–42.5	97 (25.9)	0.006	1.04 (1.01–1.08)
Pulmonary vein anatomy ^b^		n/a	9 (2.5)		
Four veins (two separate veins on the left and right side = standard)	337 (92.1)			n/a	n/a
One additional accessory vein (vs. standard)	23 (6.3)			0.056	0.48 (0.22–1.02)
Common ostium (vs. standard)	6 (1.6)			0.529	0.64 (0.16–2.58)
AAR drug use at discharge (class I or III) ^b^	171 (45.6)	n/a	0 (0.0)	0.462	1.11 (0.84–1.48)
Beta-blocker use at discharge ^b^	196 (52.3)	n/a	0 (0.0)	0.043	1.34 (1.01–1.79)
Left atrial volume (mL) ^a^ as obtained by cardiac CT	109 (90–130)	47–333	40 (10.7)	<0.001	1.01 (1.01–1.01)

Baseline characteristics (n = 375). PVI = pulmonary vein isolation, SD = standard deviation, IQR = interquartile range, BMI = body mass index, HFrEF = heart failure with reduced ejection fraction, AF = atrial fibrillation, EF = left ventricular ejection fraction, GFR = glomerular filtration rate, PAD = peripheral artery disease, RR = blood pressure, LAP = left atrial pressure, AAR = antiarrhythmic, n/a = not applicable. Mean LAP was calculated as the mean of maximum and minimum LAP. ^†^ *p*-value derived from a univariate Cox proportional hazards regression analysis considering the association of the variable with the combined endpoint. HR = crude univariate hazard ratio with a corresponding 95% confidence interval (CI). ^a^ Median value and interquartile range, ^b^ absolute numbers and percentages, ^c^ mean value and standard deviation.

**Table 2:** Different multivariate Cox proportional hazards models.

**Model 1—100 pg/mL NT-pro-BNP Increase vs. Composite Endpoint**			
**Variable**	**HR**	**95% CI**	***p*-Value**
100 pg/mL increase in NT-pro-BNP	1.04	1.01–1.07	0.005
Non-paroxysmal AF	1.83	1.16–2.87	0.009
EF (%)	1.00	0.97–1.03	0.990
BMI	1.03	0.99–1.07	0.149
GFR	1.01	1.00–1.02	0.276
Coronary artery disease	1.48	0.91–2.43	0.115
Age	0.99	0.97–1.01	0.365
Male sex	0.68	0.46–1.01	0.056
Cryoballoon generation:			
2nd vs. 1st	1.12	0.74–1.69	0.593
3rd vs. 1st	0.97	0.50–1.88	0.928
Left atrial volume	1.01	1.00–1.01	<0.001
**Model 2—NT-pro-BNP Tertile vs. Composite Endpoint**			
**Variable**	**HR**	**95% CI**	***p*-Value**
NT-pro-BNP tertile:			
2nd vs. 1st	1.21	0.76–1.91	0.417
3rd vs. 1st	1.78	1.10–2.87	0.018
Non-paroxysmal AF	1.65	1.03–2.63	0.036
EF (%)	1.00	0.97–1.03	0.910
BMI	1.03	0.99–1.07	0.150
GFR	1.01	1.00–1.02	0.218
Coronary artery disease	1.39	0.85–2.27	0.186
Age	0.99	0.97–1.01	0.317
Male sex	0.65	0.44–0.94	0.023
Cryoballoon generation:			
2nd vs. 1st	1.07	0.71–1.61	0.736
3rd vs. 1st	0.99	0.51–1.89	0.968
Left atrial volume	1.01	1.00–1.01	<0.001
**Model 3—Log NT-pro-BNP vs. Composite Endpoint**			
**Variable**	**HR**	**95% CI**	***p*-Value**
NT-pro-BNP increase of one natural log unit	1.32	1.10–1.58	0.002
Non-paroxysmal AF	1.68	1.06–2.66	0.027
EF (%)	1.00	0.97–1.03	0.911
BMI	1.03	0.99–1.07	0.164
GFR	1.01	1.00–1.02	0.153
Coronary artery disease	1.44	0.88–2.35	0.145
Age	0.99	0.97–1.01	0.175
Male sex	0.68	0.47–1.01	0.053
Cryoballoon generation:			
2nd vs. 1st	1.14	0.75–1.72	0.539
3rd vs. 1st	1.01	0.53–1.95	0.972
Left atrial volume	1.01	1.00–1.01	<0.001

Different multivariate Cox proportional hazards models considering important clinical confounders. Model 1: 100 pg/mL NT-pro-BNP increase vs. composite endpoint, Model 2: NT-pro-BNP tertile vs. composite endpoint, Model 3: natural log transformed NT-pro-BNP vs. composite endpoint. HR = hazard ratio, 95% CI = 95% confidence interval, AF = atrial fibrillation, EF = ejection fraction, BMI = body mass index.

## Data Availability

The data presented in this study are available on request. The data are not publicly available due to privacy reasons.

## Supplementary Materials

The following supporting information can be downloaded at: https://www.mdpi.com/article/10.3390/jcm11247400/s1, Figure S1: Time-dependent ROC curve; Figure S2: P-spline models for different variables.
