# Supplementary material for: NT-pro-BNP as a Predictor for Recurrence of Atrial Fibrillation after Primary Cryoballoon Pulmonary Vein Isolation
Source: J Clin Med. 2022 Dec 14;11(24):7400. doi: 10.3390/jcm11247400 (PMC9783297; doi:10.3390/jcm11247400)
Supplement: Supplementary file 1 [file jcm-11-07400-s001.zip › jcm-2038615-supplementary.pdf]

Supplementary Materials

Figure S1. Time-dependent ROC curve

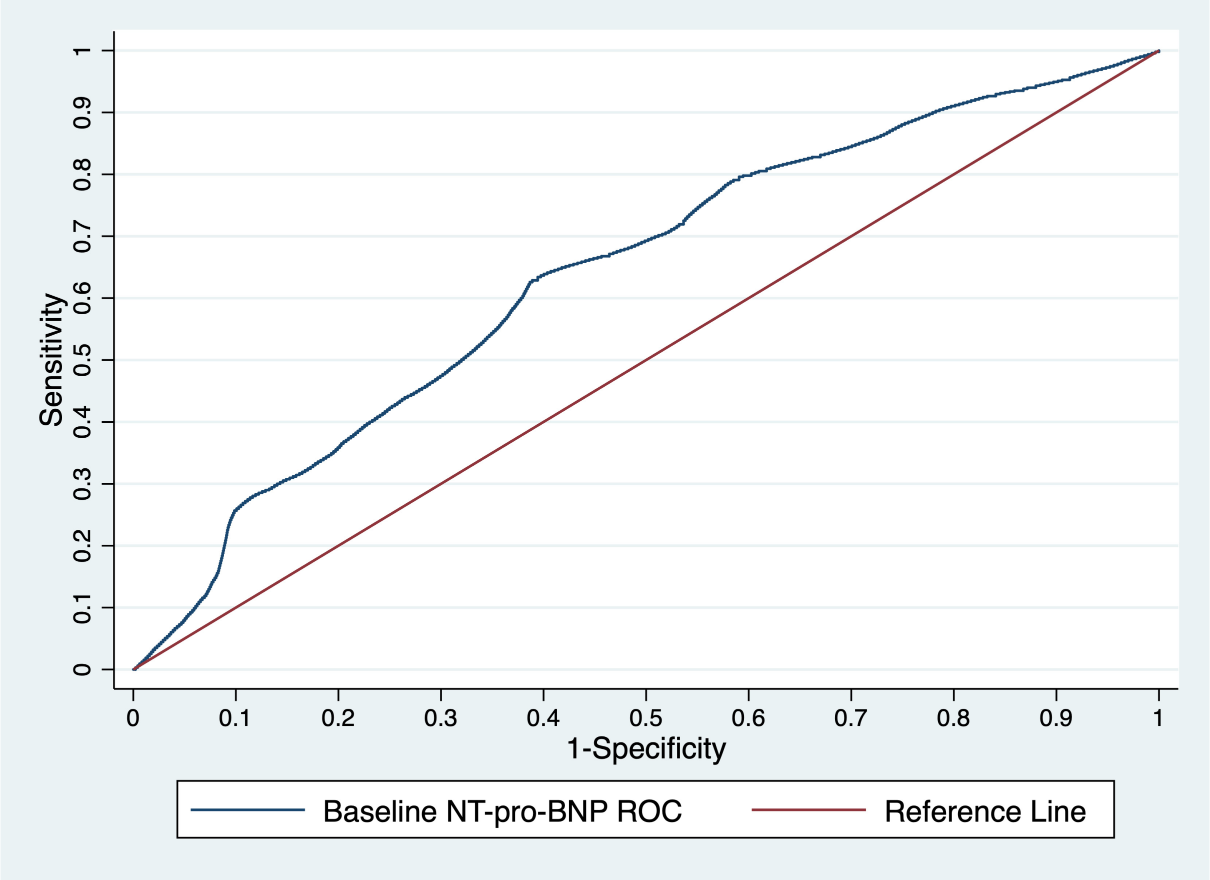

Figure S1: Time-dependent ROC curve for treatment failure (any form of AF relapse) as a function of NT-proBNP serum levels at baseline. The curve was calculated applying 0/1 nearest-neighbor kernel smoothing of the conditional survival function.

Figure S2. P-spline models for different variables.

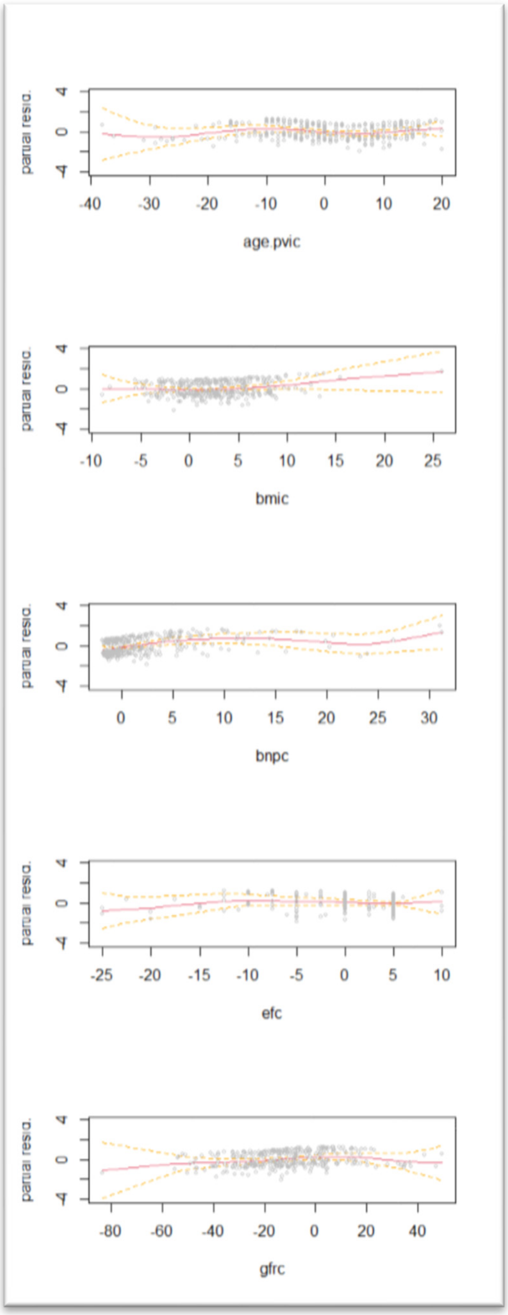

Figure S2: P-spline models investigating the possibility of non-linear effects between different variables and the composite endpoint. Grey points = data points, red line = partial regression line, dashed orange line = 95% confidence interval. Age.pvi = age at pulmonary vein isolation, bmic = body mass index, bnpc = NT-pro-BNP serum concentration, efc = left ventricular ejection fraction, gfrc = glomerular filtration rate. All variables are centered at the median.
